# Brachypodium as an experimental system for the study of stem parenchyma biology in grasses

**DOI:** 10.1371/journal.pone.0173095

**Published:** 2017-03-01

**Authors:** Jacob Krüger Jensen, Curtis Gene Wilkerson

**Affiliations:** 1 Department of Plant Biology, Michigan State University, East Lansing, MI, United States of America; 2 DOE Great Lakes Bioenergy Research Center, Michigan State University, East Lansing, MI, United States of America; 3 Department of Biochemistry and Molecular Biology, Michigan State University, East Lansing, MI, United States of America; Murdoch University, AUSTRALIA

## Abstract

Stem parenchyma is a major cell type that serves key metabolic functions for the plant especially in large grasses, such as sugarcane and sweet sorghum, where it serves to store sucrose or other products of photosynthesis. It is therefore desirable to understand the metabolism of this cell type as well as the mechanisms by which it provides its function for the rest of the plant. Ultimately, this information can be used to selectively manipulate this cell type in a controlled manner to achieve crop improvement. In this study, we show that *Brachypodium distachyon* is a useful model system for stem pith parenchyma biology. Brachypodium can be grown under condition where it resembles the growth patterns of important crops in that it produces large amounts of stem material with the lower leaves senescing and with significant stores of photosynthate located in the stem parenchyma cell types. We further characterize stem plastid morphology as a function of tissue types, as this organelle is central for a number of metabolic pathways, and quantify gene expression for the four main classes of starch biosynthetic genes. Notably, we find several of these genes differentially regulated between stem and leaf. These studies show, consistent with other grasses, that the stem functions as a specialized storage compartment in Brachypodium.

## Introduction

In many grasses, the stem parenchyma cells function as a heterotrophic tissue for the deposition of excess photosynthate for medium- to long-term storage [1–3]. This mechanism is in contrast to short-term energy reservoirs, such as ‘transitory starch’ in leaves, which allow respiration in the dark period and are adjusted for depletion at the end of the night [4]. The medium- to long-term storages however, become important under stress conditions. These stores enable the plant to fuel growth processes during less favorable growth conditions where current levels of photosynthesis cannot meet current demand for energy and carbon [5].

Medium- to long-term carbon storages may consist of fructans, sucrose, hexoses and starch. It has also been proposed that mixed-linkage glucan, in some cases such as leaf of barley seedlings, functions as a storage polymer [6]. Storage of starch and sucrose has been described in stems of numerous crops, e.g. wheat [7] and sorghum [8], as well as storage of fructans has been reported for instance in wheat and barley stems in vegetative phase and during grain filling [9, 10]. The dimensions of the stem such as width and total length are important in determining the stem reserve storage [11] and numerous quantitative trait loci and individual dwarfing genes that affect culm and stem length have been identified, e.g. the Norin 10-derived *Rht*_*1*_ and *Rht*_*2*_ in wheat [12], while more than 1000 different short-culm mutants have been identified in barley [13]. These stores in the stem contribute to gain filling and appear in particular to underpin this process during fluctuating growth conditions, such as drought, heat, or as foliar diseases tend to spread towards anthesis [10, 14, 15]. It is therefore important to gain a better understanding of source–sink relationships between stem, leaf and grain at different developmental phases.

Sugarcane represents one of the most successful examples of commercial application of grass stem parenchyma for photosynthate accumulation. Though there are indications that the system has further potential for increases in both sucrose production via photosynthesis and storage via stem parenchyma, over the recent years plant breeders have been unsuccessful in furthering increases of sucrose content in the culm. The specific reasons for the lack of progress are unresolved but evidence exists for inhibitory mechanisms that may exert this apparent roadblock, e.g. source-sink feedback signaling, end-product inhibition of photosynthesis, and/or non-optimal phloem loading and unloading processes [16, 17]. Other inefficiencies, related to specifically sucrose, are the buildup of osmotic potential by high concentration of sucrose, as well as, futile cycling (sucrose continuously being hydrolyzed by invertase and reformed by hexokinase and sucrose synthase at the expense of ATP), which each elevate the culm maintenance respiration rate [17]. In order to develop solutions to these challenges, a basic understanding of stem parenchyma cell physiology, and in particular source-sink signaling between leaf and culm, must be obtained and a biotechnological tool set must be developed to manipulate or alter these mechanisms in the right cell types, at the right developmental stage, and under the right environmental conditions.

We propose the use of *Brachypodium distachyon* as a model system for studying medium- to long-term carbon storage in heterotrophic stem pith parenchyma cells. Brachypodium is smaller in statue, has a shorter generation time and transformed plants are more readily obtained than in case of maize, sorghum or sugar cane, for instance [18, 19]. Hence, physiological investigation, tool development and testing of synthetic biology strategies will be faster and take fewer resources in a model system such as Brachypodium. The results presented here show that Brachypodium could likely be a significant aid in gaining basic understanding and improving practical application of grass stem parenchyma.

## Materials and methods

### Plant growth

Seeds of *Brachypodium distachyon* Bd21-3 were germinated in 42 x 42 mm peat pellets (Jiffy, http://www.jiffypot.com/; Product no. 70000116) and transferred to 10 x 10 x 14 cm pots with SureMix soil (Surefill, http://www.surefill.com/) after one week. In case of “stem-elongating” conditions, the environmental growth conditions were kept at 200 μEm^-2^s^-1^ light, 16-hour day and 8-hour night, 21°C day and 20°C night, and 60% relative humidity throughout, while pots were placed with no spacing in between them resulting in approximately 10 cm spacing from plant to plant. As an example of more commonly used growth chamber conditions [18, 19], a treatment of 14-day vernalization at 4°C followed by 20-hour light, 24°C; 4-hour dark, 18°C; 150 μE were applied. Under these conditions plants were placed at an approximately 20 cm spacing from plant to plant.

### Statistical analysis

The probability of significant differences between two sets of measurements was determined by the Student’s *t*-Test, assuming a two-tailed distribution and two-sample equal variance.

### Characterization of biomass accumulation

The aerial vegetative organs of 12-week-old plants were harvested in liquid nitrogen and freeze-dried for 48 hours. Dry weight of the material was determined immediately after the freeze-drying process.

### Photosynthate quantification and iodine staining

Starch and sucrose content was determined on 10 mg aliquots (three biological replicas for each condition) of freeze-dried and homogenized plant material using coupled enzyme assays from Megazyme (https://secure.megazyme.com/; Product no. K-TSTA and K-GLUC). Middle and bottom stem fractions represent stems of 12-week-old plants divided in thirds by length, the cuts being approximately at the S-02 and S-03 positions. Iodine staining was performed on 0.3 to 1.0 mm thick hand-cut sections using Lugol solution (Sigma, http://www.sigmaaldrich.com/; Product no. 32922). More than ten individual plants from several growth batches were investigated by iodine staining and showing similar results.

### Chloroplast auto-fluorescence by confocal laser scanning microscopy

Chloroplast auto-fluorescence was observed by excitation at 488 nm and collecting the 650–700 nm emission interval. Microscopy was performed using a laser confocal scanning microscope (FV1000D IM-IX81; Olympus, http://www.olympusamerica.com/) on 0.3 to 1.0 mm thick hand-cut sections from one of the main stems on three individual plants showing similar results.

### TEM analysis

For transmission electron microscopy samples were prepared as described by Turner and Somerville [20] using Poly/Bed 812 (Polysciences, http://www.polysciences.com/) as imbedding resin and viewed by transmission electron microscopy (100CX; Japan Electron Optics Laboratory, http://www.jeol.com/).

### Total RNA isolation and qRT-PCR

Total RNA was extracted from four biologically independent replica samples of stem and leaf by a modified version of a protocol developed for pine tree [21]. After extraction with the cetyltrimethyl ammonium bromide (CTAB) buffer samples were extracted twice with chloroform and precipitated with 2 M LiCl_2_. The precipitated RNA was subsequently retrieved by centrifugation and purified using the RNeasy Micro Kit and RLT buffer (74004; Qiagen, http://www.qiagen.com/) with DNase treatment (79254; Qiagen, http://www.qiagen.com/) as per manufacturer’s protocol.

Reverse transcriptase reaction was performed using 1 μg total RNA per reaction, oligo dT primer and SuperScript III Reverse Transcriptase (ThermoFischer Scientific, https://www.thermofisher.com; Product no. 18080093). qPCR was performed using Fast SYBR Green Master Mix (ThermoFischer Scientific, Product no. 4385612). S1 Table list qPCR primers used to quantify expression of selected genes.

## Results

### Mimicking the growth pattern of commercial larger grasses in Brachypodium

Growth of *Brachypodium distachyon* Bd21 under both long and short day conditions result in a short phenotype with relatively little stem material, and so shows little resemblance to the growth phenotype of larger grasses such as sugarcane, sorghum and maize grown in conventional cropping systems, where a majority of the growth phase concerns stem formation and elongation. However, we hypothesized that such a growth pattern of the commercial larger grasses could be mimicked in Brachypodium by prolonging the vegetative growth phase and by inducing internode elongation through the shade avoidance response.

**Fig 1:**
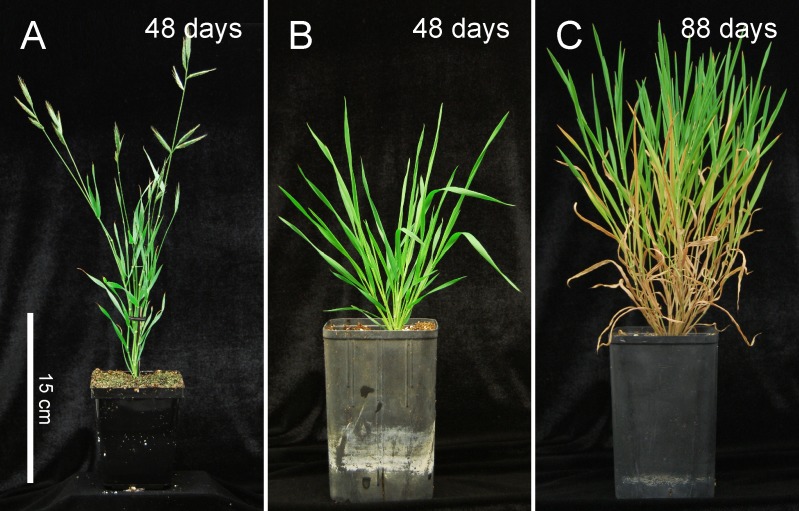
Characterization of Brachypodium growth pattern under two different conditions. (A) Brachypodium grown under more commonly used growth chamber conditions (20-hour light, 24°C; 4-hour dark, 18°C; 150 μE). (B,C) Brachypodium grown under stem-elongating conditions (16-hour light, 20°C; 8-hour dark, 21°C; 220 μE).

The degree of vernalization and length of photoperiod determine the length of the vegetative growth stage in Brachypodium and considerable variation is present between different accessions [22–25]. Among these, the inbred line Bd21 is one of the least responsive, being largely vernalization independent and only mildly affected by photoperiod, however, the closely related inbred line Bd21-3 [19] has a vernalization requirement and is more affected by photoperiod [22]. Other accessions have much stronger dependence on vernalization and photoperiod and so very long vegetative growth phases are achieved; however choosing Bd21 or Bd21-3 has the advantage that most resources in Brachypodium research pertain to these two inbred lines, e.g. the genome sequence of Bd21 [26] and the development of highly efficient transformation protocols for both [18, 19]. By subjecting Bd21-3 to a, for Brachypodium, relatively short day length (16-hour), large pot size, dense spacing, lower temperature, and omitting vernalization we obtained a prolonged vegetative growth phase and enhanced stem elongation over more than 50 days (Fig 1B and 1C; “stem-elongating” conditions). Under more commonly used growth chamber conditions, including vernalization and a longer photoperiod [18, 19], Bd21-3 flowers after 30 days (Fig 1A), whereas under our modified stem-elongating conditions, plants remain vegetative for as much as 100 days and produce a large amount of elongated stem material (Fig 1B and 1C).

**Fig 2:**
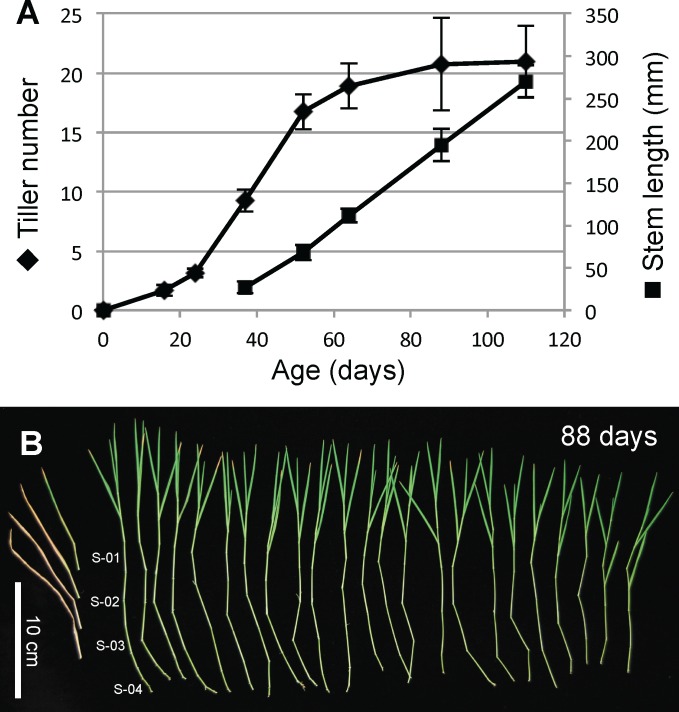
Characterization of Brachypodium growth pattern under stem-elongating conditions. (A) Number of tillers and length of main stem as a function of plant age. Measurements were performed on three independent batches of plants with similar results whereof one is shown. Standard deviation is shown (n = 16). (B) Diagram showing all stems of a single plant and location of four internodes sampled along the stem, S-01 to S-04. Senescent leaves were removed. The senescent leaves for the main stem are placed at the left.

To characterize the growth pattern of Bd21-3 under these conditions, we quantified tiller number and length of the primary tiller as a function of plant age (Fig 2A). This analysis revealed that after a period of establishment of about 20 days, tiller production is prolific for 30–60 days, resulting in about 20 tillers per plant, while after this period few new tillers emerge. At about day 40 we observe an increased rate of stem elongation, which continues, in a linear fashion until the onset of flowering. These growth characteristics result in tillers with several highly elongated internodes, typically 2–3 cm in length but occasionally longer (Fig 2B). Another characteristic of these plants is that the lower leaves senesce (Figs 1C and 2B), leaving each stem with 1–2 mature green leaves and several developing younger leaves as part of the shoot. The senescence of the lower leaves is also seen for commercially grown sugarcane, sorghum and maize [3, 17] and as such represents an additional characteristic that this model system shares with crop plants.

To facilitate our analysis, we define four internode locations or stages of the 12-week-old plants, designated S-01 to S-04. Stage S-01 consists of the top non-elongating internode and stage S-04 consists of the internodes formed in the transition to stem elongation at day 30–40 and are approximately 1 cm long. Stage S-02 and S-03 are spaced equally along the stem between stage S-01 and S-04.

We measured the mass of stem and leaf for Bd21-3 plants grown for 12 weeks under stem-elongating conditions (Table 1). Of the tissues stem and green leaf, the stem portion exerts about 40% of wet weight biomass, with the same water content as the green leaf portion (*t*-test, r = 0.24). A comparison of the dry weight of senescent and green leaves shows that about half of the total leaf material is senescent at the 12-week stage.

**Table 1:** Biomass accumulation in aerial vegetative organs of 12-week-old plants.

	Wet weight (g)^1^	Dry weight (g)^1^^,^^2^	Water content (%)
Stem	1.8 ± 0.1	0.74 ± 0.06 *	58
Green leaf	2.9 ± 0.2	1.07 ± 0.08	62
Senescent leaf	0.65 ± 0.06	0.54 ± 0.04 *	17

^1^Standard error, n = 8.

^2^Dry weight significantly different from green leaf (t test)

*, <0.01.

### Brachypodium stem pith parenchyma functions as a carbon storage compartment

**Fig 3:**
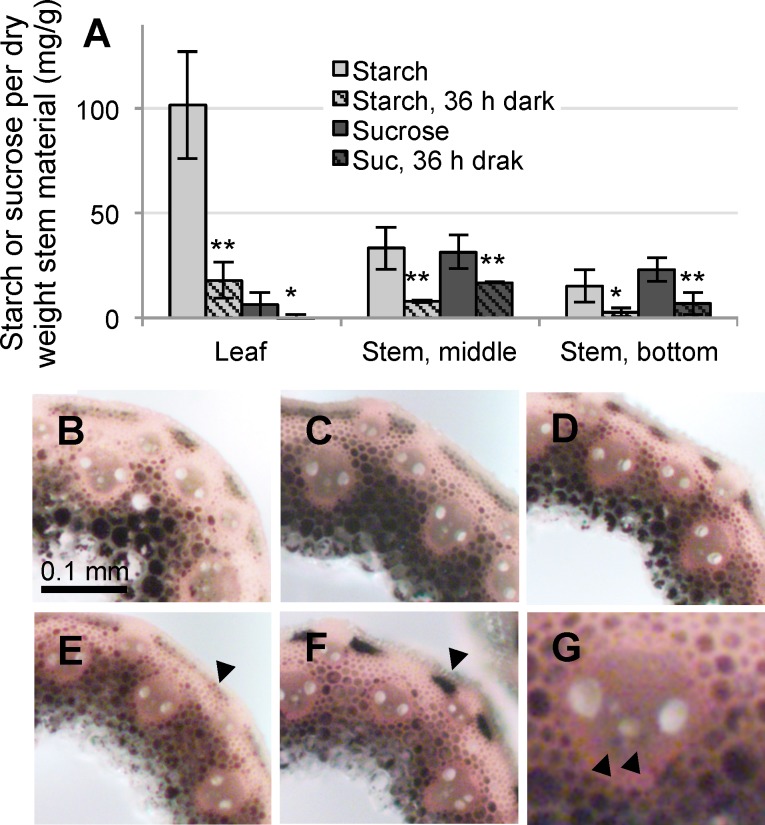
Carbon storage in the Brachypodium stem. (A) Quantification of starch and sucrose levels in leaf, middle part of stem, and bottom part of stem, with and without dark-treatment. (B-G) Iodine staining of Brachypodium stem cross-sections. Progressive stem stages from bottom towards the top of the stem are shown (B, S-04; C, S-03; D, S-02; E and F, S-01). E is from within while F is from above the leaf sheath. More starch is accumulated in the cortex parenchyma in F compared to E (arrow) while the starch levels in the pith parenchyma appear equal in the two samples. G shows iodine staining of starch granules in parenchyma cells in the vascular bundle (arrows).

To assess the degree of stored carbon in the stem we measured the amounts of starch and sucrose for the middle and bottom thirds of the stem and compared it to green leaf (Fig 3A). Plants were harvested at two time points, at the end of the light cycle and after a dark period of 36 hours. This analysis shows that significant amounts of both starch and sucrose are stored in the stem and that these energy resources can be mobilized during a prolonged period of dark. A conservative estimation based on Table 1 and Fig 3A reveals that at least 20% of the starch and sucrose of the aerial part of the plants (represented by stem and green leaf) measured at the end of the light period is stored in the stem (see S1 File for detailed calculation).

Cells in the stem storing starch were identified by iodine staining of stem sections at S-01 to S-04. Areas of dark staining with iodine indicate starch accumulation. At each location sections showed extensive staining of dark granules, particularly within the pith parenchyma cells of the stem (Fig 3B–3F). Some dark staining was also found in the vascular bundles (Fig 3G), the innermost interfascicular fiber cells, and in pockets of chloroplast containing parenchyma cells in the cortex. At this latter location, strong stain appeared in sections from internodes taken above the leaf sheath (Fig 3F), which was reduced when compared to below the leaf sheath (Fig 3E). Contrarily, starch accumulation at other locations, i.e. pith, interfascicular and vasculature, was independent of internode position compared to leaf sheath.

### Chloroplast pigmentation and thylakoid membrane stacks alter with development and location within the stem

**Fig 4:**
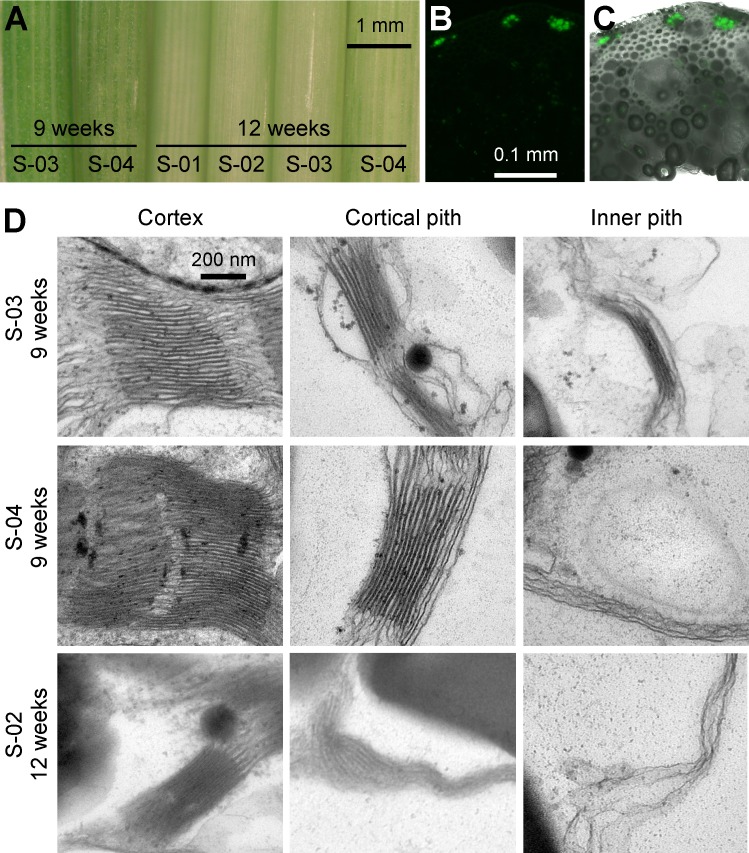
Plastid development in the Brachypodium stem. (A) Decrease of stem chlorophyll in stem from 9- to 12-week-old plants. (B) Chloroplast auto-fluorescence by confocal laser scanning microscopy. Cross-section is equivalent to section in Fig 3F (S-01 internode, above leaf sheath, 12-week-old plant). (C) Merged image of auto-fluorescence in (B) with the bright field channel showing that the strong auto-fluorescence originates from cortex parenchyma cells. (D) TEM analysis of thylakoid membrane stacks in internodes of plants of different age and at different locations within the stem cross-section.

We observed that stem color changed with the age of the plant. As seen in Fig 4C, internodes at position S-03 and S-04 for 9-week-old plants are visibly greener than at any of the internode positions for 12-week-old plants. These observations lead us to characterize the morphology of thylakoid membrane stacks by TEM for plastids at different locations in the stem for internode positions S-03 and S-04 in 9-week-old plants, and S-02 in 12-week-old plants (Fig 4D; for TEM micrographs of lower magnification see S1 and S2 Figs). The 9-week S-03 internode is equivalent to the 12-week S-02 internode in age in terms of how recently they were formed at the time of harvest. In agreement with overall chloroplast pigmentation in the cortex; the number and stacking depth of thylakoid membrane stacks were reduced between the 9- and 12-week time points. A similar progression was observed for cortical and inner pith located plastids, however these showed much-reduced thylakoid membrane stacks even for S-03 at 9-week-old plants. Hence, for S-02 in 12-week-old plants no thylakoid membrane stacks were observed for inner pith located plastids. In agreement with these observations we also found that cortex parenchyma have much higher auto-fluorescence compared to any of the other cell types (Fig 4B and 4C). Based on the lack of thylakoid membrane stacks, lack of chlorophyll fluorescence, and the prominent occurrence of starch granules, the plastids of the pith parenchyma cells can be categorized as amyloplasts [27].

### Differential expression of genes involved in starch biosynthesis between stem and leaf

In higher plants, four classes of enzymes, i.e. ADP-glucose pyrophosphorylase, starch synthase, starch branching enzyme, and starch debranching enzyme, are essential for starch biosynthesis. ADP-glucose pyrophosphorylase performs a key function in directing photosynthate towards starch accumulation. The other three classes of enzymes are involved in determining starch primary structure, as well as granule size, shape and crystallinity [28]. In Brachypodium, 21 different genes encode the four classes of starch producing enzymes [29].

To gain further insights into starch biosynthesis in the stem compared to green leaf in Brachypodium we compared the expression levels of the four classes of genes involved in starch biosynthesis (Table 2) along with the expression levels of five housekeeping genes. This analysis revealed that genes encoding three of the four starch biosynthetic enzyme classes examined were highly differentially expressed in stem versus leaf. Genes showing high expression in stem and low expression in leaf included ADP-glucose pyrophosphorylase small subunit (Bradi4g27570), ADP-glucose pyrophosphorylase large subunit 3 (Bradi2g14970), starch synthase IIa (Bradi1g45130), starch synthase granule-bound I (Bradi1g50090), and starch branching enzyme IIb (Bradi3g44760). On the contrary, the genes Bradi1g09537 and Bradi2g41590, encoding ADP-glucose pyrophosphorylase large subunit 1 and starch synthase granule-bound IIb, respectively, are each expressed ten times higher in leaves than in stem. Hence, in case of ADP-Glucose pyrophosphorylase large subunit, Brachypodium has two genes inversely regulated between stem and leaf. A remaining third homolog, ADP-glucose pyrophosphorylase large subunit 4, shows similar but low levels of expression in both tissues. For the granule-bound starch synthase enzyme class, Brachypodium has two homologs, i.e. the ones just mentioned. Hence, in the case of ADP-glucose pyrophosphorylase large subunit and granule-bound starch synthase the partitioning between stem and leaf in the form of close homologs being inversely expressed is pronounced. The remaining genes are expressed in both stem and leaf at comparable levels showing a few fold differences. These findings suggest that pith parenchyma tissues have plastids that differ from those in leaves.

**Table 2:** Expression of starch related genes in stem and leaf.

Gene	Annotation	Normalized expression^1^^,^^2^		Stem / leaf^3^
		Stem	Leaf	
**ADP-Glucose pyrophosphorylase**				
Bradi4g27570	Small subunit, AGP-S	2.0 ± 0.3	0.2 ± 0.1	9**
Bradi3g22330	Small subunit, AGP-S	0.23 ± 0.04	0.37 ± 0.06	0.6**
Bradi1g53500	Large subunit, BdAPL4	0.00002 ± 0.00001	0.000027 ± 0.000004	0.5
Bradi2g14970	Large subunit, BdAPL3	2.5 ± 1.3	0.002 ± 0.001	1284**
Bradi1g09537	Large subunit, BdAPL1	0.05 ± 0.01	0.71 ± 0.08	0.1**
**Starch synthase**				
Bradi1g48610	SSI	0.44 ± 0.07	0.7 ± 0.2	0.7
Bradi1g45130	SSIIa	0.062 ± 0.013	0.0013 ± 0.0004	48**
Bradi3g59440	SSIIb	0.016 ± 0.003	0.016 ± 0.004	1
Bradi3g27260	SSIIc	0.046 ± 0.006	0.07 ± 0.01	1**
Bradi3g15027	SSIIIa	0.0017 ± 0.0005	0.0008 ± 0.0002	2*
Bradi5g22310	SSIIIb	0.04 ± 0.01	0.10 ± 0.02	0.4**
Bradi2g18810	SSIV	0.10 ± 0.01	0.09 ± 0.02	1
Bradi1g50090	Granule-bound, GBSSI	0.65 ± 0.05	0.0006 ± 0.0004	1075**
Bradi2g41590	Granule-bound, GBSSIIb	0.031 ± 0.007	0.27 ± 0.04	0.1**
**Starch branching enzyme**				
Bradi1g41970	SBEIII	0.018 ± 0.004	0.05 ± 0.01	0.3**
Bradi5g09170	SBEIIa	1.7 ± 0.3	0.7 ± 0.2	3**
Bradi3g44760	SBEIIb	0.004 ± 0.003	0.00004 ± 0.00002	97**
Bradi1g29850	SBEI	2.16 ± 0.06	0.4 ± 0.2	7**
**Isoamylase**				
Bradi2g26170	ISA2	0.057 ± 0.005	0.038 ± 0.009	1*
Bradi3g40410	ISA1	0.13 ± 0.01	0.04 ±0.01	3**
Bradi4g32707	ISA3	0.09 ± 0.02	0.040 ± 0.005	2**
**Housekeeping genes**				
Bradi1g17970	60S ribosomal protein L18-3-like	0.33 ± 0.02	0.20 ± 0.05	2**
Bradi3g30710	Actin-3-like	1.0 ± 0.1	0.8 ± 0.2	1
Bradi3g49600	Protein PEROXIN-4-like	0.14 ± 0.02	0.12 ± 0.02	1
Bradi3g58226	Polyubiquitin-A-like	13 ± 2	9 ± 2	2*
Bradi0012s00200	Elongation factor 1-alpha-like	8.3 ± 0.7	4 ± 1	2**

^1^Expression normalized to expression of housekeeping gene Actin-3-like (Bradi3g30710) in stem.

^2^Standard error, n = 4.

^3^Expression level between stem and leaf is significantly different (*t* test)

*, <0.05 and

**, <0.01.

## Discussion

We show that Brachypodium can be used as a model for the study of grass stem physiology and development in general. By manipulating growth conditions, an extended vegetative period can be obtained where tiller formation has essentially ceased and continual growth occurs only by formation of additional internodes and corresponding leaves. Under these conditions, the stem portion continues to increase compared to the green leaf portion, as leaf senescence is balanced with the new leaf formation, resulting in a constant level of 2–3 adult green leaves per stem. Hence, for 12-week-old plants the stem portion comprises approximately 40% of the aerial green tissue of the plant. From cross-sections (Fig 3B–3G) it is evident that half or more of the stem cross-area represent cell types that show starch accumulation, i.e. in cortical, vascular, cortical pith, and inner pith parenchyma. The collective stem parenchyma cells thereby represent 20% or more of the aerial tissues at the 12-week time point indicating that this tissue represents a sink tissue of significant capacity.

We find that 3.8 percent or more of the stem dry weight is composed of starch and sucrose (S1 File). These stores occur fairly evenly throughout the different internodes, regardless of whether the leaf associated with the particular internode is senescent or not. The plant utilizes these carbon stores during extended dark conditions (Fig 3A), even in the oldest internodes, showing that the stem parenchyma is metabolically active.

Starch accumulated mainly in the stem cortex in regions of internodes extending above the leaf sheath. It appears that these parenchyma cells have significant photosynthetic capacity, evident by chloroplast auto-fluorescence and abundant thylakoid membrane stacks, while the plastids in the pith parenchyma in older plants resemble amyloplasts; as these have few or no thylakoid membrane stacks, no chloroplast auto-fluorescence, and prominent starch granules. The cortex parenchyma constitutes a smaller fraction of the stem tissue than pith parenchyma, hence the bulk of carbon stores in the stem likely originate from sucrose supplied by another organ producing excess fixed carbon, whereof leaf is a likely possibility.

The specialized function of the stem as a carbon storage compartment is reflected by the differential regulation of starch synthesis genes between stem and leaf. Several of these genes are found expressed higher in stem than in leaf, while in all cases a close homolog is present that is expressed in both stem and leaf at comparable levels or at higher levels in leaf. The latter group could be associated with transitory starch synthesis in photosynthetic cells, including stem cortical parenchyma, and the former associated with storage in heterotrophic cells. The most abundantly expressed stem specific starch genes encode the ADP-glucose pyrophosphorylase small (Bradi4g27570) and large (Bradi2g14970) subunits, the latter being expressed more than 1000 fold higher in the stem than leaves. These enzymes regulate the metabolic flux into the starch synthesis pathway by catalyzing the first enzymatic step in the pathway. Accordingly, these have been selected as target genes for up regulation in cereals (maize, rice, wheat) and dicots (potato and cassava) with the aim to increase starch accumulation in seed or tuber [30–32]. In Brachypodium these genes therefore likely serve an important role in starch accumulation in the stem.

The isolation of promoter fragments that specifically drive gene expression in stem pith parenchyma would be of great value as a biotechnological tool. Genes such as Bradi2g14970 (ADP-glucose pyrophosphorylase large subunit 3), which display high expression in the stem and not in leaf, are likely associated with cis regulatory elements that confer such desired expression characteristics, and so such genes serve as target regions for further investigations.

Engineering grasses to accumulate hexoses or other compounds in the pith parenchyma cells at high levels would likely provide improved bioenergy crops. To address these and other objectives we need a more comprehensive understanding of stem parenchyma metabolism, primarily through metabolomics and transcriptomics in both applied and model systems. The Brachypodium growth conditions for Bd21-3 described here are a prerequisite to obtaining enough stem material for some of these analysis, while at the same time, having the experimental advantages of a model system.

## Supporting information

S1 Fig Lower magnification of thylakoid membrane TEM analysis. (PDF)

S2 Fig Higher magnification of thylakoid membrane TEM analysis. (PDF)

S1 File Calculation of total amount of photosynthate deposited in stem. (DOCX)

S1 Table qRT-PCR primer list. (DOCX)
