# Supplementary material for: Brachypodium as an experimental system for the study of stem parenchyma biology in grasses
Source: PLoS One. 2017 Mar 1;12(3):e0173095. doi: 10.1371/journal.pone.0173095 (PMC5332097; doi:10.1371/journal.pone.0173095)

# S1 Fig

**Supplementary Figure S1. TEM of thylakoid membrane structure and starch granules.**

Specific tissue location of micrographs is indicated in the top panel, and age and location along the stem of the section is indicated in the left side panel.

Scale bar in each picture is 0.4 µm.

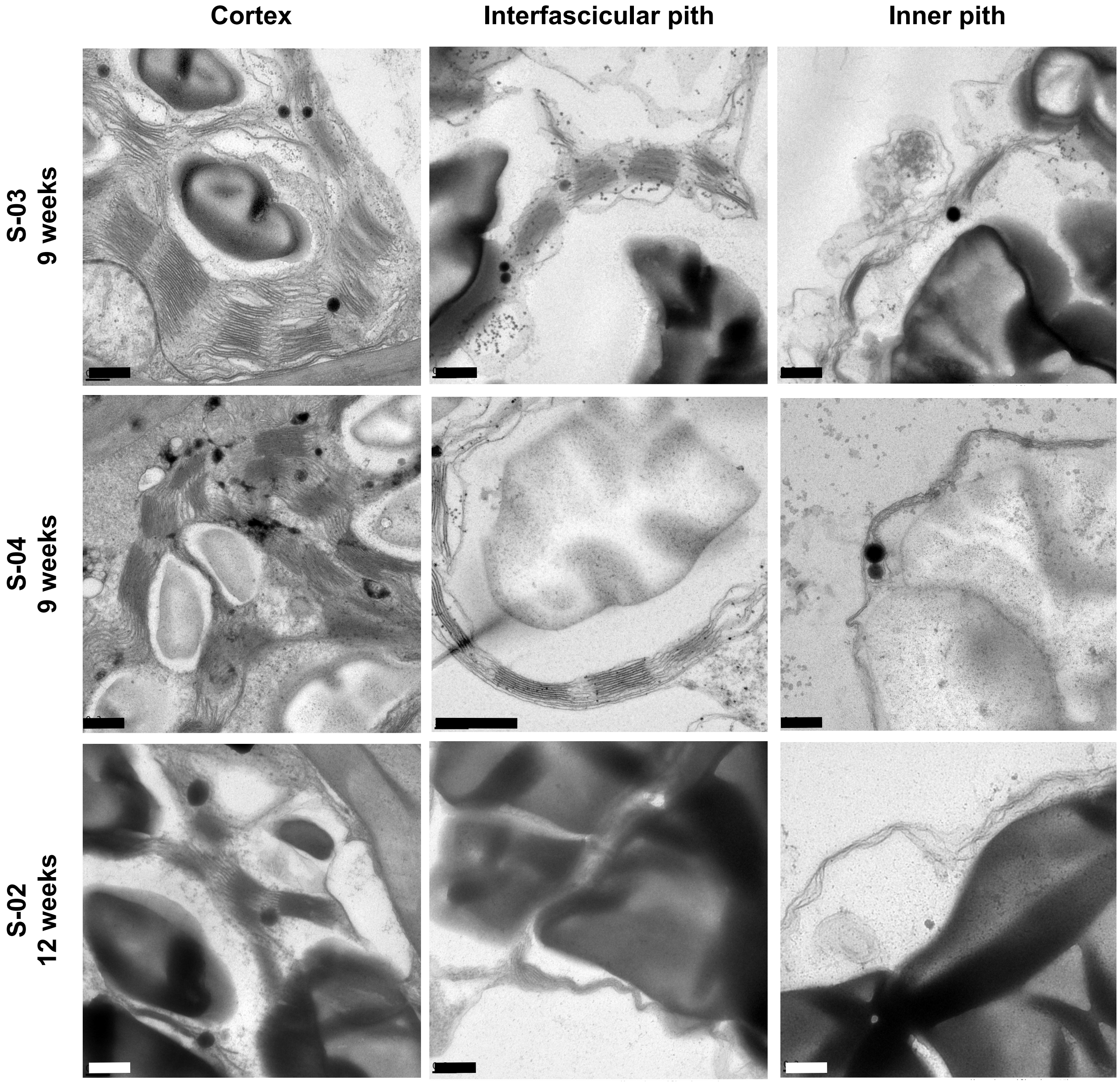

Supplement: S1 Fig — (PDF) [file pone.0173095.s001.pdf]
