# Supplementary material for: Brachypodium as an experimental system for the study of stem parenchyma biology in grasses
Source: PLoS One. 2017 Mar 1;12(3):e0173095. doi: 10.1371/journal.pone.0173095 (PMC5332097; doi:10.1371/journal.pone.0173095)

S2 Fig

Supplementary Figure S2. TEM at whole chloroplast to whole cell level.

Specific tissue location of micrographs is indicated in the top panel, and age and location along the stem of the section is indicated in the left side panel.

Scale bar in each picture is 2 μm.

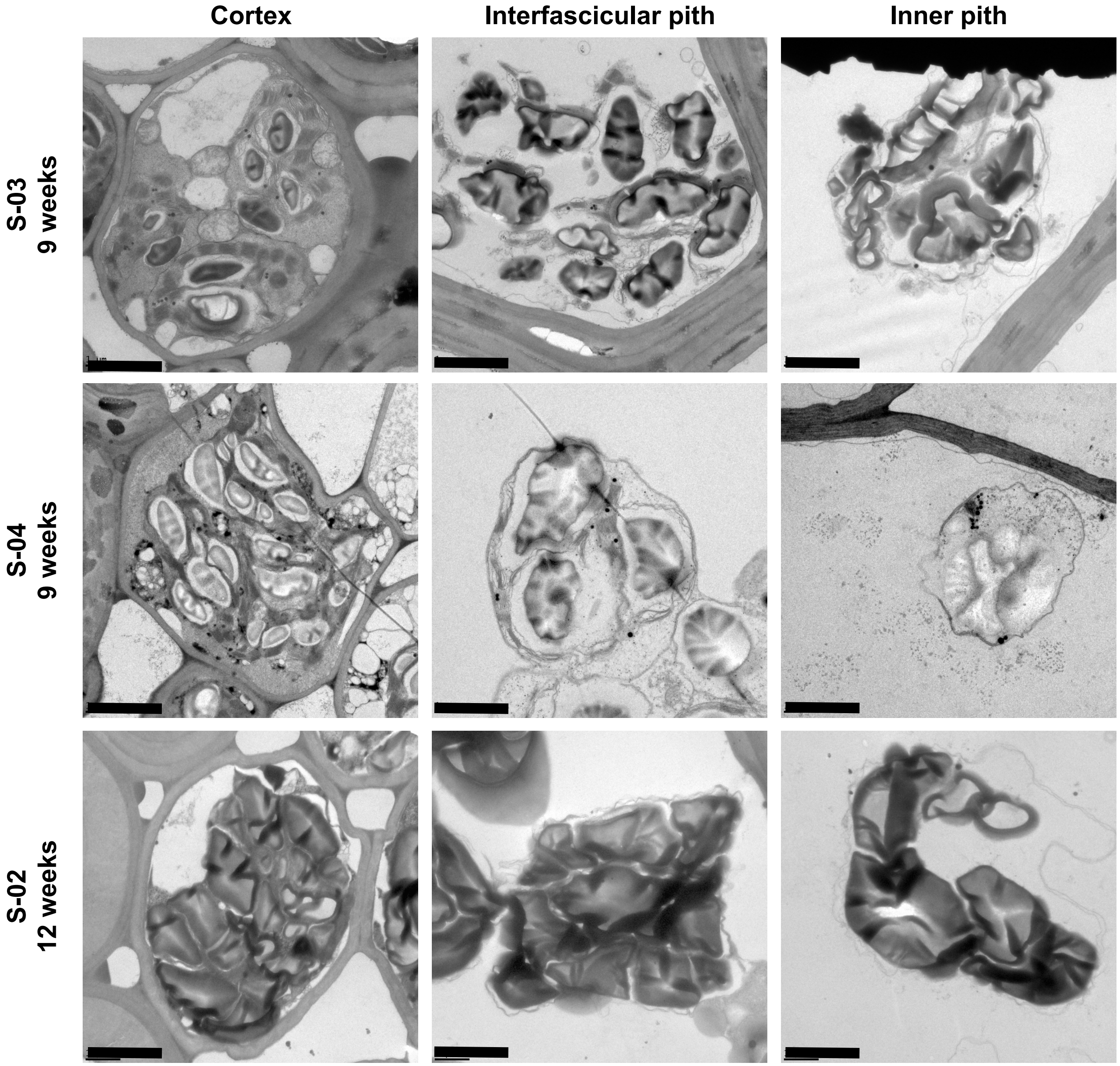

Supplement: S2 Fig — (PDF) [file pone.0173095.s002.pdf]
