# Supplementary material for: Brachypodium as an experimental system for the study of stem parenchyma biology in grasses
Source: PLoS One. 2017 Mar 1;12(3):e0173095. doi: 10.1371/journal.pone.0173095 (PMC5332097; doi:10.1371/journal.pone.0173095)
Supplement: S1 File — (DOCX) [file pone.0173095.s003.docx]

**S1 File. Calculation of total amount of photosynthate deposited in stem**

**Total starch and sucrose per plant in green leaf:**

Based on Figure 3A the combined starch and sucrose levels in leaf at the end of the photoperiod is approximately 100 mg per 1 g of dry weight leaf material. Table 1 shows that a 12-week old plant has 1.07 g of dry weight green leaf material. Hence, approximately 107 mg starch and sucrose is allocated per plant in green leaf (1.07 g dry weight material per plant x 100 mg/g starch and sucrose = 107 mg starch and sucrose per plant).

**Total starch and sucrose per plant in stem:**

Based on Figure 3A the combined starch and sucrose levels of the mid and bottom stem sections are approximately 70 and 45 mg/g, respectively. The average of this is 57.5 mg/g starch and sucrose for the lower two thirds of the stem. The starch and sucrose levels were not quantified for the top third of the stem. Assuming no starch and sucrose in the top section and equal mass of plant material of the three sections produce 38 mg/g. This number represent the lower limit for the total starch and sucrose content of the whole stem. This is a conservative estimate as iodine staining shows the presence of starch granules throughout the whole stem (Fig. 3B-G).

Table 1 shows that a 12-week old plant has 0.74 g of dry weight stem material. Hence, approximately 28 mg starch and sucrose is allocated per plant in the stem (0.74 g dry weight material per plant x 38 mg/g starch and sucrose = 28 mg starch and sucrose in the stem per plant).

Combining the amounts of starch and sucrose from green leaf and stem yields 135 mg per plant (107 mg + 28 mg = 135 mg).

The relative amount of starch and sucrose allocated in the stem is therefore at least 20% of the total amount for stem and green leaf (28 mg / 135 mg = 20.7 %).
