# Supplementary material for: Brachypodium as an experimental system for the study of stem parenchyma biology in grasses
Source: PLoS One. 2017 Mar 1;12(3):e0173095. doi: 10.1371/journal.pone.0173095 (PMC5332097; doi:10.1371/journal.pone.0173095)
Supplement: S1 Table — (DOCX) [file pone.0173095.s004.docx]

**S1 Table. qRT-PCR primer list**

| **ADP-Glucose pyrophosphorylase** | |
| --- | --- |
| Bradi4g27570 | AAT AAC TGC GCC CTC TGA TAT G |
|  | AGG TTC TTG ACG CTG ATG TG |
| Bradi3g22330 | CCC ATG AGT AAT GTG TCC TCT ATT |
|  | CGG CGA AGG GTG TGT TAT TA |
| Bradi1g53500 | TGA GCG GAT ACC AAT AAC AGA G |
|  | CTC CAA CCA AGG TGG AAG AT |
| Bradi2g14970 | TAC CCT TCC TCT GCC CTA TC |
|  | CAA CTG CAT CAT CGA CAT GAA C |
| Bradi1g09537 | AAT CGT CGT GTT CTC TCC TAT TC |
|  | CTG TAA TGC TTG GTG CTG ATT T |
| **Starch synthase** |  |
| Bradi1g48610 | TTC ATT AGC CCT TCC CAA GAC |
|  | GGG CAT TCT CAC CAC TAA CA |
| Bradi1g45130 | ATC CGC TAC CGC TGT TTC |
|  | ACC GGA ACT ACA GGG AGA G |
| Bradi3g59440 | CAC TGG TAC TTG GCC TTG A |
|  | CCT CAA CAC GTA CTG GAA CTA C |
| Bradi3g27260 | CCC TTC CAC AGC TTT CAT GTA G |
|  | TTT GCC CAG TAT CCC AGT TC |
| Bradi3g15027 | CTG TCA GCT CCG TTG AAA CT |
|  | GGG CTC TAT GAT ACG GTC TTT G |
| Bradi5g22310 | GTG TCA TAA AGG CCT CCA GTT |
|  | TAT GCT GGT GCC GAC TTT ATT |
| Bradi2g18810 | GCT GCT GCT CTT TGG TAG AT |
|  | CGA GGT CTG GAA ACA GTT AGT G |
| Bradi1g50090 | ATG CAG TTC CTG ACC ATC TC |
|  | CGA GAC TGG GTT CCA CAT G |
| Bradi2g41590 | CAC ATT TTG TTT CGC AAG TGG |
|  | AGA ATT GTA TGG CTC AGG ACC |
| **Starch branching enzyme** | |
| Bradi1g41970 | TGC GTG AGT ATG GAC ATT GG |
|  | CGA AAC TCC TTG GAA TTG ACA TTA G |
| Bradi5g09170 | GTC TAA GGC CAC CTT GTA CTT C |
|  | CTT CGA GAG AGG AGA TTT GGT ATT T |
| Bradi3g44760 | CCC ACA CGG TAG TCG AAA TAG |
|  | CGG AAA CAC GAG GAG GAT AAG |
| Bradi1g29850 | ATC CTC TGT TGA CGC CTT TC |
|  | CTG GGA AGA TTG CTC TGG ATT AT |
| **Isoamylase** |  |
| Bradi2g26170 | GCA CTT GTT GCA CTT TAG GAT TAG |
|  | CAG AAG GAT CCA TGT GGC TAT G |
| Bradi3g40410 | GCG ATA CAA GGA TGA TGG AAG A |
|  | TTC AGC ATC CAC CAG TTC TC |
| Bradi4g32707 | CTT TCC ATG TTC TCC CAA CAA TC |
|  | TCG CCA GAA TCA CCC AAT AC |
| **Housekeeping genes** | |
| Bradi1g17970 | CCT TCC TCT TGC CTT CTC AAA |
|  | AAT GCC AGG GAA GCT GTT AG |
| Bradi3g30710 | TGC TCA TAC GGT CAG CAA TAC |
|  | TGA GAC CAC CTA CAA CTC TAT CA |
| Bradi3g49600 | GTA GAG GAT ACT GCT CAG GAA TTG |
|  | ATT CAA GTG GAC GGC TCT AAT C |
| Bradi3g58226 | GGA CTC CTT CTG GAT GTT GTA G |
|  | GAC CAG CAG CGT CTC ATA TT |
| Bradi0012s00200 | GAT GAT GAC CTG TGA GGT GAA G |
|  | TGT TGC TGT GAA GGA TCT CAA |
